# Comparative Phosphoproteomics of Two *Flammulina filiformis* Cultivars with Differential Postharvest Browning Susceptibility

**DOI:** 10.3390/jof12020115

**Published:** 2026-02-05

**Authors:** Yu Fu, Xiaojing Guo, Xiaolan He, Bo Wang, Weihong Peng, Yang Yu

**Affiliations:** 1 College of Pharmacy and Food, Southwest Minzu University, Chengdu 610225, China; yufu@swun.edu.cn (Y.F.); guoxiaojing1923@163.com (X.G.); 2 National-Local Joint Engineering Laboratory of Breeding and Cultivation of Edible and Medicinal Fungi, Sichuan Institute of Edible Fungi, Sichuan Academy of Agricultural Sciences, Chengdu 610000, China; xiaolanhe1121@aliyun.com (X.H.); 4504292@163.com (B.W.); pwh424@163.com (W.P.)

**Keywords:** Phosphoproteomics, *Flammulina filiformis*, Energy deficiency, postharvest deterioration, ATP level

## Abstract

Protein phosphorylation modification plays a role in cells’ response to oxidative stress, a key factor leading to postharvest browning of *Flammulina filiformis*. However, the molecular mechanism by which protein phosphorylation contributes to postharvest browning of *F. filiformis* remains unclear. This study aimed to characterize the basal phosphoproteomic landscapes associated with variations in different browning phenotypes of *F. filiformis*. Using data-independent acquisition (DIA) mass spectrometry, we comprehensively profiled the phosphorylation dynamics in susceptible-to-browning (SB) and resistant-to-browning (RB) cultivars at harvest and after 24 h storage. We identified 84,244 phosphorylation sites on 4494 phosphoproteins, with the SB cultivar displaying more altered sites (21,195) than the RB (16,087). Functional enrichment analysis revealed that the differential phosphorylation was significantly implicated in kinases and energy metabolism pathways. Notably, the SB cultivar exhibited a more pronounced phosphorylation profile on key proteins involved in ATP synthesis and glycolysis. Protein–protein interaction (PPI) network analysis further indicated a kinase-mediated regulatory network targeting core energy metabolism components, including ATP synthase and 6-phosphofructokinase. This distinct phosphosignature in the SB cultivar correlated with its more severe browning phenotype and a sharper decline in ATP content during storage. Our findings suggest that divergent phosphorylation-mediated regulation of energy metabolism is strongly associated with the differential postharvest browning susceptibility between these two cultivars, providing a valuable molecular resource for future functional studies.

## 1. Introduction

*Flammulina filiformis* (golden needle mushroom) is a widely cultivated and highly valued edible fungus, prized globally for its nutritional profile, distinctive texture, and flavor [1]. It holds particular commercial significance in East Asia, with China being the world’s foremost producer and consumer, driving a major agricultural industry dedicated to its cultivation and postharvest management [1,2]. The sustained market demand for fresh, high-quality *F. filiformis* underscores the importance of extending its postharvest shelf life.

The postharvest preservation of *F. filiformis* is critical for maintaining its commercial value, yet significant quality deterioration, including rapid browning, remains a major challenge. Notably, some cultivars typically exhibit far more severe and rapid postharvest browning compared to others, leading to greater economic losses [3,4]. This deterioration is primarily driven by oxidative stress, which triggers a cascade of events including enzymatic browning, membrane lipid peroxidation, and cellular senescence [4,5]. Enzymatic browning, in particular, involves the oxidation of endogenous phenolic compounds to quinones, which subsequently polymerize into melanin [6]. While this oxidative damage pathway is well-recognized, the fundamental molecular determinants that predetermine the differential browning susceptibility between susceptible-to-browning (SB) and resistant-to-browning (RB) cultivars are still largely unexplored.

Oxidative stress-induced reactive oxygen species (ROS) can modify the activity of protein kinases and phosphatases by oxidizing specific amino acid residues, including cysteine and methionine. For example, ROS can oxidize and activate kinases within the mitogen-activated protein kinase (MAPK) signaling pathway, thereby promoting downstream substrate phosphorylation. This can lead to the activation of signaling pathways associated with cell cycle arrest and apoptosis [7]. Previous studies have shown that genes related to the MAPK signaling pathway (MPK3, MPK6, and PR1a1) are significantly induced within 10 min of chitosan treatment, effectively reducing postharvest decay in cherry tomatoes [8]. This finding was further confirmed in chitosan-treated papaya, where similar results were observed [9]. More recently, it was discovered that caffeic acid-grafted chitosan/polylactic acid (CA-g-CS/PLA) packaging can upregulate key genes in the MAPK signaling pathway of *Agaricus bisporus*, such as Sho1, Ssk2, Pbs2, and Hog1. This upregulation facilitates the accumulation of stress-resistant compounds and delays postharvest quality deterioration [10]. These results collectively demonstrate that ROS generated under oxidative stress directly interacts with protein kinases and phosphatases, playing a crucial role in maintaining the postharvest quality of fruits and vegetables.

Protein phosphorylation also plays a significant role in the cellular response to oxidative stress. It can regulate the activity of antioxidant enzymes, such as glutathione peroxidase (GPx), by phosphorylating these enzymes and enhancing their ROS-scavenging capacity, thereby mitigating oxidative stress damage [11]. In addition, protein phosphorylation is involved in modulating various oxidative stress-related signaling pathways, including the NF-κB and Nrf2 pathways. For instance, phosphorylation can activate NF-κB, promoting its nuclear translocation and subsequent induction of antioxidant enzyme expression, which in turn regulates cellular responses to oxidative stress [11]. Therefore, protein phosphorylation and oxidative stress interact closely, playing vital roles in helping horticultural crops and edible fungi cope with oxidative stress and maintain cellular homeostasis postharvest.

Protein phosphorylation is not only involved in the regulation of plant responses to abiotic stress but also in the development and progression of diseases in animals and humans, particularly those associated with oxidative stress, inflammation, and aging [12,13]. In these disease processes, altered phosphorylation patterns have been observed in proteins involved in cellular signaling pathways, including those related to stress response, apoptosis, and metabolic regulation [14]. Dysregulation of phosphorylation signaling pathways is often linked to diseases such as cancer, where aberrant activation of protein kinases drives tumorigenesis [12]. Therefore, understanding the functional and regulatory mechanisms of protein phosphorylation is essential for elucidating both physiological and pathological states. Previous transcriptomic and proteomic analyses have revealed that genes and proteins related to the oxidative phosphorylation pathway are induced during the browning process of *F. filiformis* [4]. We therefore hypothesize that divergent phosphorylation-mediated signaling underlies the distinct postharvest physiological trajectories of different mushroom cultivars. A comprehensive, phosphoproteome-wide comparison could reveal the specific kinase–substrate networks and metabolic pathways that are differentially regulated between slow-browning and fast-browning varieties, offering unprecedented molecular insights into their inherent quality traits.

To test this hypothesis, we conducted a comparative phosphoproteomic analysis of SB and RB cultivars of *F. filiformis* at harvest and after 24 h storage. This study aims to characterize the basal phosphoproteomic landscapes of these two cultivars and identify the key phosphorylation-regulated processes that correlate with their differential browning phenotypes. Rather than focusing solely on oxidative stress mechanisms, our work seeks to map the foundational molecular differences that may govern energy metabolism, stress signaling, and ultimately, postharvest resilience. By providing the first detailed phosphoproteomic resource for *F. filiformis*, this study lays the groundwork for understanding the molecular basis of postharvest quality and identifies potential targets for future breeding or biochemical intervention strategies.

## 2. Materials and Methods

### 2.1. Sample Preparation

*F. filiformis* SB strain Chuanjin No. 3, with distinct postharvest browning characteristics, and a cultivar, Chuanjin No. 11, generated through hybrid breeding from Chuanjin No. 3, characterized by its reduced postharvest browning, was included as an RB cultivar in this study [5]. They have similar agronomic traits. Both strains were bred by the Sichuan Institute of Edible Fungi. The samples used in this study were obtained from industrialized cultivation, where mature and uniformly growing *F. filiformis* were harvested in their entirety from the medium, with one plant serving as one biological replicate. Each strain was sampled with five plants as biological replicates, and the fresh fruiting bodies were transported back to the laboratory in a foam box containing ice packs. Fruiting bodies were stored unsealed at room temperature (25 °C) for 24 h. Samples were collected both before storage (0 h postharvest, 0 hph) and after storage (24 h postharvest, 24 hph). The designations “0 hph” and “24 hph” represent the fruiting body samples before and after the 24 h storage period, respectively.

### 2.2. Protein Extraction and Quality Tests

Samples were ground in liquid nitrogen and lysed with SDS-DTT-Tris buffer (SDT buffer) (BIOESN, Shanghai, China) containing 100 mM NaCl and 1/100 volume dithiothreitol (Sigma-Aldrich, St. Louis, MO, USA). After 5 min of ultrasonication on ice, samples were incubated at 95 °C for 10 min, then cooled in an ice bath for 2 min. Centrifugation was carried out at 12,000 *g* for 15 min at 4 °C using a centrifuge (Eppendorf, Hamburg, Germany), after which the supernatant was collected. Iodoacetamide (Thermo Fisher Scientific, Waltham, MA, USA) was added to the supernatant for a 1 h dark reaction at room temperature. Samples were mixed with 4 volumes of precooled acetone (Sinopharm Chemical Reagent, Shanghai, China), incubated at -20 °C for 2 h, then centrifuged again (12,000 *g*, 4 °C, 15 min). The precipitate was collected, washed with cold acetone, and dissolved in dissolution buffer (DB).

For protein quantification via Bradford assay, bovine serum albumin (BSA) standard solutions (Bio-Rad, Hercules, CA, USA) and diluted sample solutions were added to a 96-well plate. After adding G250 dye (Pierce Coomassie Plus Assay Kit, Thermo Fisher Scientific) and incubating, absorbance at 595 nm was measured using a microplate reader (BioTek Synergy H1, Winooski, VT, USA) to construct a standard curve (linear range: 0–0.6 μg/μL, coefficient of determination (R^2^): 0.9908) and calculate sample protein concentration.

In SDS-PAGE, 20 μg protein samples were loaded onto a 12% gel. Electrophoresis was run at 80 V (stacking gel, 20 min) and 120 V (running gel, 90 min). Samples were then stained and destained until protein bands were visible.

### 2.3. Trypsin Treatment

Protein samples were adjusted to 100 μL with DB lysis buffer (8 M urea, 100 mM TEAB, pH 8.5). After adding trypsin (Roche Diagnostics GmbH, Mannheim, Germany) and TEAB buffer, the mixture was vortexed and digested at 37 °C for 4 h, followed by an overnight digestion with additional trypsin. Formic acid was then added to lower the pH to below 3, and the sample was centrifuged at 12,000 *g* for 5 min. The supernatant was loaded onto a Sep-Pak Vac C18 solid-phase extraction cartridge (Waters, Milford, MA, USA), washed three times, and eluted. The eluted fractions were combined and lyophilized.

Chromatography began with isocratic elution (96% mobile phase A, 4% B) at a decreasing flow rate (2.5–0.8 nL/min) from 0 to 0.5 min. Gradient elution followed (0.5–21.5 min), increasing mobile phase B while decreasing A for effective component separation. A column wash occurred at 21.5 min, and re-equilibration restored the initial mobile phase composition by 22.6 min.

### 2.4. LC-MS/MS Analysis—DIA Mode

Mobile phase A (0.1% formic acid in water) and mobile phase B (0.1% formic acid in 80% acetonitrile) were prepared. The lyophilized powder was dissolved in 10 μL of mobile phase A, centrifuged at 14,000 *g* (4 °C, 20 min), and 200 ng of the supernatant was injected for liquid quality detection.

A Vanquish Neo UHPLC system (Thermo Fisher Scientific, Waltham, MA, USA) with a heated C18 pre-column (174,500, 5 mm × 300 μm, 5 μm, Thermo Fisher Scientific) at 50 °C and a C18 analytical column (ES906, PepMap™ Neo UHPLC, 150 μm × 15 cm, 2 μm, Thermo Fisher Scientific) was used.

A Thermo Orbitrap Astral mass spectrometer (Thermo Fisher Scientific, Waltham, MA, USA) with an Easy-Spray (ESI) ion source was employed. The ion spray voltage was 2.0 kV, and the ion transfer tube temperature was 290 °C. The mass spectrometer operated in DIA mode with a full-scan MS range of *m*/*z* 380–980. The primary MS resolution was 240,000 (at *m*/*z* 200), AGC target 500%, precursor ion window 2 Th, DIA windows 300, and NCE 25%. The secondary *m*/*z* range was 150–2000 with a sub-ion resolution of 80,000 and a maximum injection time of 3 ms. Raw data were saved in .raw format.

### 2.5. The Identification and Quantification of Protein

The raw data files were subjected to database-assisted searching and analysis using the DIA-NN library search software (version 1.8.1) and the UniProt protein database. The library search parameters were configured as follows: a mass tolerance of 10 ppm for precursor ions and 0.02 Da for fragment ions. Cysteine residues were modified via alkylation, methionine residues underwent oxidative modification, and N-terminal modifications included acetylation, loss of methionine, and loss of methionine plus acetylation. Up to two missed cleavage sites were permitted.

To enhance the quality of the analytical results, the DIA-NN software further filtered the search results by retaining only credible peptide spectrum matches (PSMs) with a confidence level of 99% or higher. Only credible spectral peptides and proteins were retained, and false discovery rate (FDR) validation was performed to exclude peptides and proteins with an FDR exceeding 1%. Proteins with a fold change (FC) greater than or less than a specified threshold value were defined as differentially regulated phosphoproteins (DPPs). Retention time correction was performed using internal retention time (iRT) markers added to the samples, and the precursor ion Q-value cutoff was set at 0.01.

### 2.6. The Functional Analysis of Protein and DPPs

Gene Ontology (GO) and InterPro (IPR) functional analyses were performed using the InterProScan program against a non-redundant protein database, including Pfam, PRINTS, ProDom, SMART, ProSite, and PANTHER [15]. Additionally, the databases of COG (Clusters of Orthologous Groups) and KEGG (Kyoto Encyclopedia of Genes and Genomes) were utilized to analyze protein families and pathways. DPPs were subjected to volcano plot analysis, cluster heatmap analysis, and enrichment analysis of GO, IPR, and KEGG pathways [16].

### 2.7. Statistical Analysis

All statistical analyses were performed using SPSS 26.0 (IBM). Data are presented as mean ± SEM. Group differences were evaluated by Student’s *t*-test, with *p* < 0.05 defining significance and |log_2_FC| > 1 defining differential protein expression. Graphs were created using GraphPad Prism 8.0.

## 3. Results

### 3.1. Differential Protein Phosphorylation Profile of SB and RB Cultivars Before and After Storage

**Figure 1:**
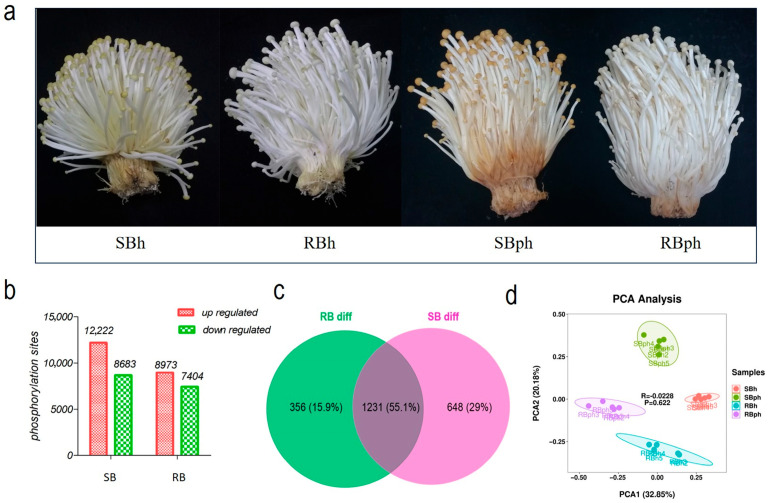
Characterization of phosphorylation in *F. filiformis* during postharvest storage. (**a**) Visual degradation (discoloration and deterioration) in SB and RB *F. filiformis* fruiting bodies pre- and post-24 h storage. (**b**) upregulated and downregulated phosphorylation sites in two cultivars. (**c**) Venn diagram of shared phosphorylation sites with significant changes between two cultivars during storage. (**d**) Principal component analysis (PCA) for all samples. SBh, susceptible-to-browning phenotype at 0 h; RBh, resistant-to-browning phenotype at 0 h; SBph, susceptible-to-browning phenotype after 24 h postharvest; RBph, resistant-to-browning phenotype after 24 h postharvest.

The SB cultivar developed a significantly more pronounced browning phenotype at the base than the RB cultivar after 24 h of storage (Figure 1a). To profile the phosphoproteome, data-independent acquisition (DIA) mass spectrometry was employed to analyze the fruiting bodies of both cultivars at 0 and 24 h postharvest (hph).

The analysis identified a total of 31,937 peptides, corresponding to 4494 phosphoproteins and 84,244 phosphorylation sites. Comparative analysis revealed that in the SB cultivar (SBh vs. SBph), 20,905 phosphorylation sites were significantly altered after storage, comprising 12,222 downregulated and 8683 upregulated sites. In the RB cultivar (RBh vs. RBph), 16,377 sites were significantly altered, with 8973 downregulated and 7404 upregulated (Figure 1b).

A Venn diagram analysis of differentially phosphorylated proteins (DPPs) showed that 1587 DPPs were identified in the RB cultivar (RBh vs. RBph), while 1879 were identified in the SB cultivar (SBh vs. SBph). Among these, 356 DPPs were unique to the RB cultivar, 648 were unique to the SB cultivar, and 1231 DPPs overlapped between the two cultivars during postharvest progression (Figure 1c). Principal component analysis (PCA) of the phosphorylation data demonstrated high intra-group consistency and clear separation between the experimental groups (Figure 1d).

### 3.2. Comparative Functional Enrichment Analysis Between Two Cultivars During Storage

**Figure 2:**
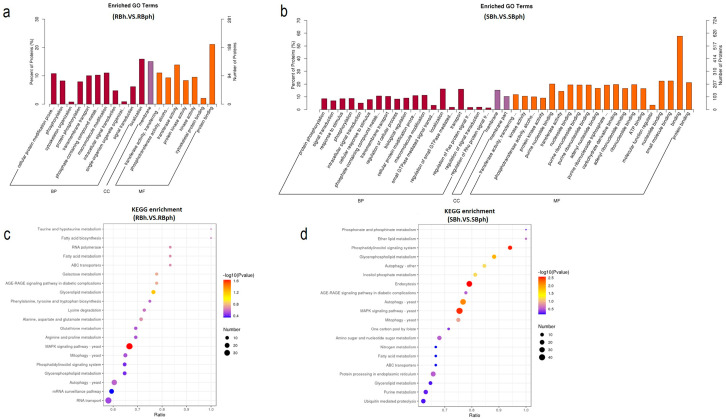
Functional annotation of differential protein phosphorylation in two cultivars. (**a**) Top enriched GO pathways in RB cultivar samples before versus after storage. (**b**) Top enriched GO pathways in SB cultivar samples before versus after storage. All proteins were classified by GO terms based on their biological process, cellular component, and molecular function. (**c**) Top enriched KEGG pathways in RB cultivar samples before versus after storage. (**d**) Top enriched KEGG pathways in SB cultivar samples before versus after storage. SBh, susceptible-to-browning phenotype at 0 h; RBh, resistant-to-browning phenotype at 0 h; SBph, susceptible-to-browning phenotype after 24 h postharvest; RBph, resistant-to-browning phenotype after 24 h postharvest.

GO enrichment analysis of DPPs in RB and SB *F. filiformis* demonstrated the key pathways by which different cultivars regulate postharvest browning. In total, 32 pathways were specifically enriched in the SB cultivar during storage (Figure 2a,b). It reveals the important role of protein phosphorylation in regulating the postharvest browning of *F. filiformis* mushrooms. In particular, several important browning regulation pathways are included in the specific pathways of the SB cultivar, such as protein phosphorylation, response to stimulus, and ATP binding (Figure 2b).

KEGG enrichment analysis of cultivar-specific phosphorylated proteins demonstrated significant divergence. Specifically, the SB cultivar exhibited specific enrichment in five pathways (*p* < 0.05), including endocytosis, the phosphatidylinositol signaling system, autophagy, and glycerophospholipid metabolism, while the RB cultivar showed only one uniquely enriched pathway (Figure 2c). The MAPK signaling pathway emerged as the sole common pathway shared by both cultivars (Figure 2d). Notably, the SB-specific pathways, particularly endocytosis, phosphatidylinositol signaling, and glycerophospholipid metabolism, displayed strong associations with membrane dynamics and oxidative stress responses, suggesting their potential mechanistic roles in browning regulation.

### 3.3. Differential Expression Analysis Between Two Cultivars During Storage

**Figure 3:**
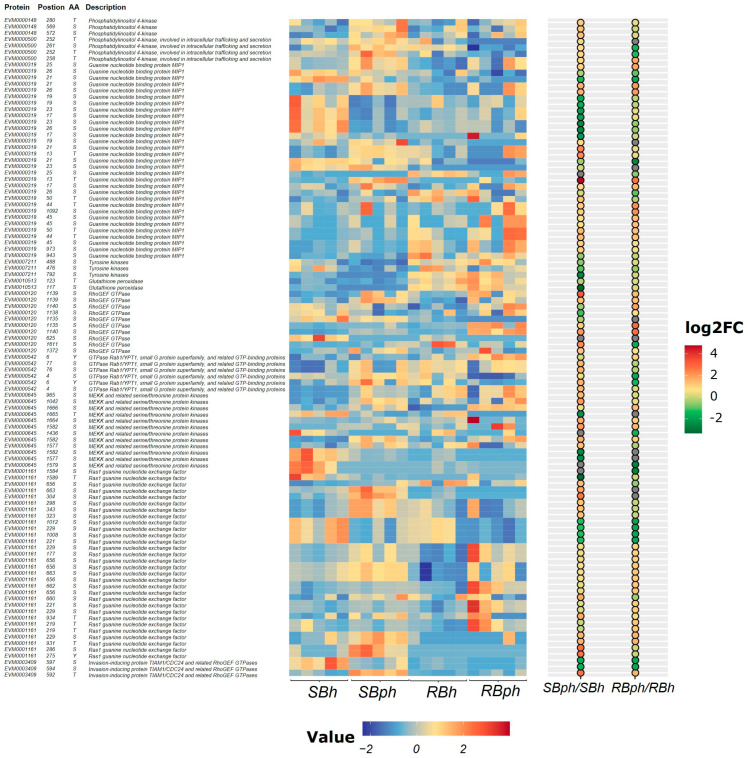
Heatmap of differential phosphoproteins potentially related to browning from enriched GO and KEGG pathways for two cultivars before versus after storage. SBh, susceptible-to-browning phenotype at 0 h; RBh, resistant-to-browning phenotype at 0 h; SBph, susceptible-to-browning phenotype after 24 h postharvest; RBph, resistant-to-browning phenotype after 24 h postharvest.

Proteins and their phosphorylation sites selected from significantly enriched pathways are presented in Figure 3. These included phosphatidylinositol 4-kinase, guanine nucleotide-binding protein MIP1, tyrosine kinases, glutathione peroxidase, RhoGEF GTPase, GTPase Rab1/YPT1, MEKK and related serine/threonine protein kinases, Ras1 guanine nucleotide exchange factor, and invasion-inducing protein TIAM1/CDC24 and related RhoGEF GTPases (Figure 3).

The SB cultivar exhibited significantly higher phosphorylation levels of phosphatidylinositol 4-kinase type 2 (PI4KII) compared to the RB cultivar. Phosphorylation levels of tyrosine kinases increased following storage in both cultivars, with a greater magnitude of increase observed in the SB cultivar. For glutathione peroxidase, phosphorylation at Ser117 increased post-storage in the RB cultivar but decreased in the SB cultivar, while phosphorylation at Thr123 decreased in both cultivars (Figure 3).

Phosphorylation at multiple sites on the Ras1 guanine nucleotide exchange factor was significantly upregulated after storage. Notably, phosphorylation at the Ser1024 residue increased 27-fold in the SB cultivar. Similarly, phosphorylation at multiple MAPK sites was significantly upregulated, with a specific site (Ser1024 on a MAPK protein) showing a nine-fold increase in the SB cultivar post-storage. Furthermore, phosphorylation levels at multiple sites on RhoGEF GTPase proteins and the abundance of invasion-inducing protein TIAM1/CDC24 and related RhoGEF GTPases were significantly higher in the SB cultivar compared to the RB cultivar after storage (Figure 3).

### 3.4. Identification and Profiling of Phosphoproteins Involved in Energy Metabolism

**Figure 4:**
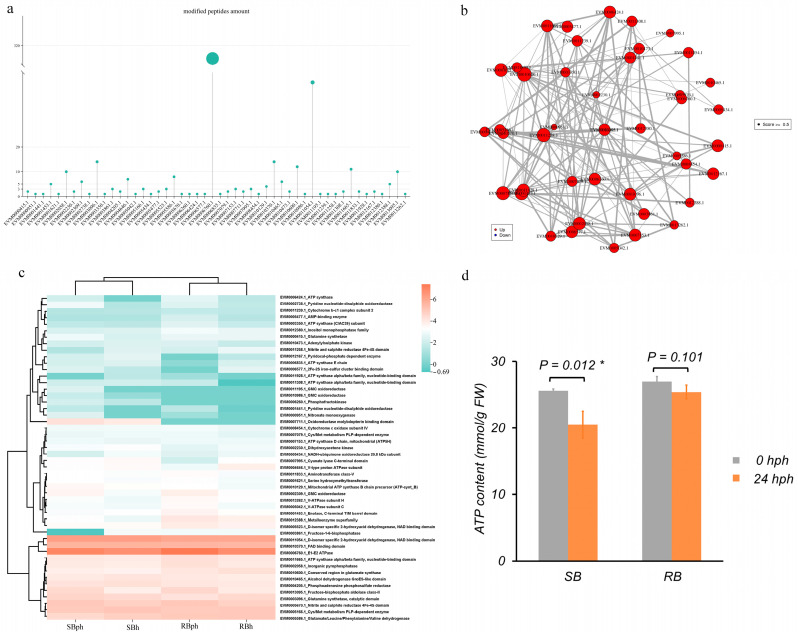
Phosphorylated proteins associated with energy metabolism and their interaction networks. (**a**) Number of peptides associated with 50 phosphorylated proteins. (**b**) Interaction network of the 50 phosphorylated proteins. (**c**) Heatmap representation of the phosphorylated proteins. (**d**) ATP content in SB and RB cultivars at 0 and 24 h postharvest, asterisks (*) denote statistically significant differences (*p* < 0.05). SBh, susceptible-to-browning phenotype at 0 h; RBh, resistant-to-browning phenotype at 0 h; SBph, susceptible-to-browning phenotype after 24 h postharvest; RBph, resistant-to-browning phenotype after 24 h postharvest.

In this study, 50 phosphorylated proteins associated with energy metabolism were identified. Among them, EVM0006760.1 (an E1-E2 ATPase) contained the highest number of peptides (370), followed by EVM0011054.1 (a D-isomer-specific 2-hydroxyacid dehydrogenase) with 46 peptides (Figure 4a).

The protein–protein interaction (PPI) network of these 50 proteins is shown in Figure 4b. Within this network, EVM0001453.1 (enolase) interacted with 23 proteins, EVM0011258.1 (nitrite and sulfite reductase) with 21 proteins, and EVM0002058.1 (inorganic pyrophosphatase) with 19 proteins. Several other proteins, including two ATP synthase alpha/beta family subunits (EVM0011665.1 and EVM0011928.1), also served as central nodes, each interacting with more than 10 partners (Figure 4b).

The phosphorylation profiles of these energy metabolism-related proteins revealed discernible differences between the two cultivars (Figure 4c). For instance, EVM0007711.1 (oxidoreductase) was phosphorylated specifically in the SB cultivar. Multiple other proteins, including EVM0011054.1 (D-isomer specific 2-hydroxyacid dehydrogenase), EVM0006760.1 (E1-E2 ATPase), and EVM0013095.1 (fructose-bisphosphate aldolase), were among the highly phosphorylated peptides across the datasets.

Furthermore, measurement of ATP content showed a significant decrease in the SB cultivar after 24 h of storage, from approximately 27 mmol/g FW at 0 hph to 21 mmol/g FW at 24 hph (Figure 4d, *p* = 0.012). In contrast, the RB cultivar maintained stable ATP levels, with no significant difference between 0 hph (~27 mmol/g FW) and 24 hph (~25 mmol/g FW) (Figure 4d, *p* = 0.101).

### 3.5. Phosphorylation Kinase Prediction Between Two Cultivars During Storage

**Figure 5:**
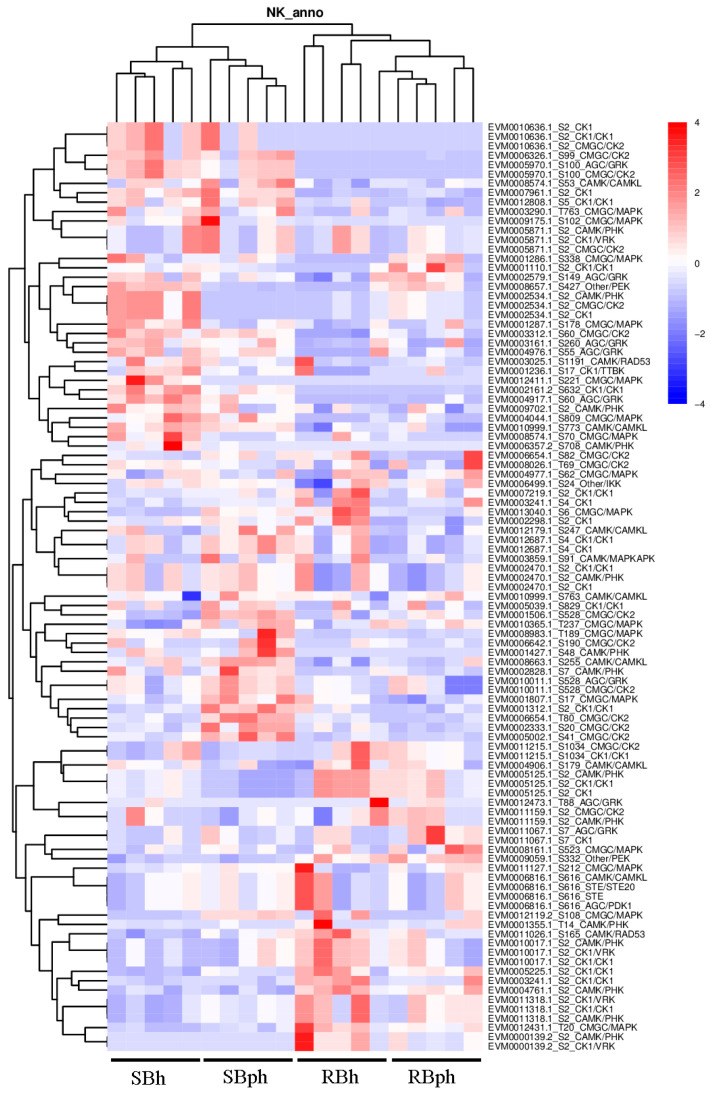
Kinase-specific phosphorylation sites and quantification of the corresponding peptides across sample groups, analyzed by GPS (group-based prediction system). SBh, susceptible-to-browning phenotype at 0 h; RBh, resistant-to-browning phenotype at 0 h; SBph, susceptible-to-browning phenotype after 24 h postharvest; RBph, resistant-to-browning phenotype after 24 h postharvest.

To identify potential upstream regulators responsible for the observed phosphoproteomic changes, we performed a comprehensive in silico prediction of protein kinases based on the identified phosphorylation sites. The top 100 kinases with the highest prediction scores were selected for comparative abundance analysis between the SB and RB cultivars (Figure 5).

Kinase classification revealed that these top predicted kinases encompassed several major families, including CMGC (encompassing CDK, MAPK, GSK3, and CLK), AGC (including GRK), CAMK/CAMKL, and CK1 (Figure 5). Quantitative analysis of kinase abundance delineated distinct cultivar-specific patterns. A significant subset of kinases demonstrated markedly higher abundance in the SB cultivar. This group included specific members of the CMGC/CK2 family (EVM0010636.1, EVM0006654.1, EVM0002333.1, EVM0005002.1), the AGC/GRK family (EVM0005970.1), the CAMK/CAMKL family (EVM0008574.1), the CK1/CK1 family (EVM0012808.1), and the CMGC/MAPK family (EVM0003290.1) (Figure 5).

Furthermore, a second cohort of kinases, while present across groups, exhibited their highest expression levels specifically in the SB cultivar. Notably, this cohort included kinases with dual family annotations or specific functional implications, such as EVM0002828.1 (CAMK/PHK), EVM0010011.1 (annotated as both AGC/GRK and CMGC/CK2), EVM0001807.1 (CMGC/MAPK), and EVM0001312.1 (CK1/CK1) (Figure 5). The overrepresentation of these diverse kinase families in the SB cultivar highlights a fundamentally altered signaling landscape potentially driving its distinct postharvest physiology.

### 3.6. In Silico Protein–Protein Interaction Network of Predicted Kinases and Energy Metabolism-Related Proteins

To explore the potential regulatory relationships between the predicted kinases and the identified energy metabolism-related proteins, a protein–protein interaction (PPI) network was constructed in silico based on database evidence. The analysis was restricted to high-confidence interactions (confidence score > 900).

**Figure 6:**
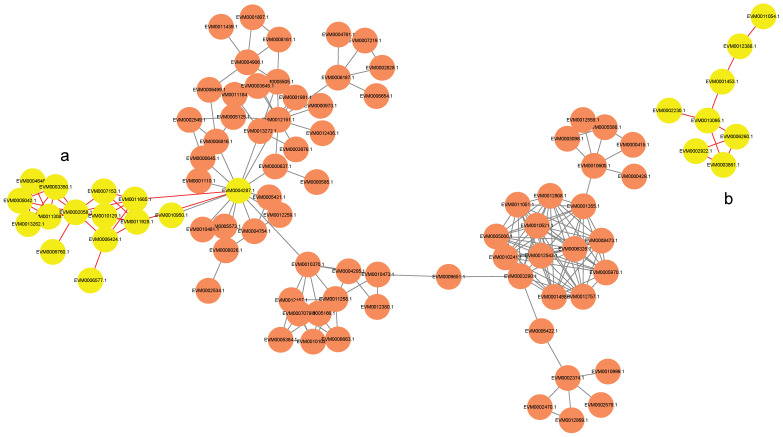
Interaction network between kinases (orange circles) and energy-related proteins with scores above 900. Yellow circles denote the target proteins in the (**a**) CAMK/ATP synthase/ATPase/PPase/SQR and (**b**) CK1/PFP/FBPase-1 pathways.

The resulting predicted network revealed putative kinase–target interactions (Figure 6). For instance, the kinase CAMK (EVM0004287) was predicted to potentially interact with subunits of mitochondrial ATP synthase (EVM0007153.1, EVM0011665.1), an E1-E2 ATPase (EVM0006760.1), an inorganic pyrophosphatase (EVM0002058.1), and a subunit of succinate–coenzyme Q reductase (EVM0006577.1) (Figure 6a). Similarly, the kinase CK1 (EVM0002922.1) was found to have predicted interactions with key glycolytic enzymes, specifically 6-phosphofructokinase (EVM0006260.1) and fructose-1,6-bisphosphatase (EVM0003861.1) (Figure 6b).

## 4. Discussion

### 4.1. Phosphoproteomic Remodeling Correlates with Browning Susceptibility

The postharvest physiological divergence between the SB and RB cultivars of *F. filiformis* was immediately evident from the significantly more pronounced browning in the SB cultivar (Figure 1a). Our phosphoproteomic analysis revealed that this phenotypic disparity is underpinned by substantial molecular differences. The SB cultivar exhibited a significantly greater magnitude of change in its phosphoproteome after storage (20,905 altered sites) compared to the RB cultivar (16,377 altered sites) (Figure 1b). This more pronounced phosphoproteomic remodeling in the SB cultivar is correlated with its severe browning phenotype. It may reflect a more extensive rewiring of cellular signaling networks upon postharvest stress. While such a global increase in phosphorylation changes has been associated with stress responses in other perishable produce, like blueberries [17], it has more recently been demonstrated in table grapes, where it modulates quality traits such as browning and senescence [18]. Our data now extend this paradigm to edible fungi and critically highlight that not just the occurrence but also the magnitude of this phosphoproteomic remodeling is a key molecular feature distinguishing the browning-susceptible cultivar.

The identification of a larger number of cultivar-specific differentially phosphorylated proteins (DPPs) in the SB cultivar (648 DPPs) compared to the RB cultivar (356 DPPs) (Figure 1c) indicates that these unique phosphorylation events are closely correlated with its accelerated browning. This aligns with the concept that genetic background shapes postharvest resilience through predefined regulatory networks [19]. Furthermore, the clear separation of groups by PCA (Figure 1d) underscores that the phosphorylation signatures are distinct for both genetic cultivar and postharvest status. Collectively, these findings highlight the potential of protein phosphorylation to serve as a key regulatory layer in the browning process of *F. filiformis*, consistent with its established role as a rapid switch for cellular signaling in plant and fungal stress responses [8,9].

### 4.2. Association Between Altered Signaling Networks and the Browning Phenotype

The SB cultivar exhibited more pronounced phosphorylation changes on multiple signaling proteins, including tyrosine kinases, as well as dramatic upregulation on the Ras1 guanine nucleotide exchange factor (Ras1 GEF; 27-fold at Ser1024) and MAPK proteins (Figure 3). The co-occurrence of these alterations suggests a coordinated perturbation within interconnected signaling networks during storage.

These observations invite interpretation within a broader biological context. It is noteworthy that the signaling components showing differential phosphorylation in our study, such as those in the Ras/MAPK cascade and tyrosine kinase-mediated pathways, are evolutionarily conserved regulators of critical cellular processes. For instance, Ras/MAPK signaling is a fundamental module governing cell growth and stress adaptation across kingdoms [20], while tyrosine kinase pathways are central to complex trait regulation, including pigment synthesis in diverse systems [21]. In the context of postharvest physiology, the MAPK cascade is a well-established central signaling module that transduces stress signals, including those initiated by reactive oxygen species (ROS), to orchestrate cellular responses, including senescence [7]. The established importance of these pathways lends biological plausibility to our observation that their phosphorylation state is a major point of divergence between the cultivars. Therefore, the coordinated phosphorylation changes we detected represent a compelling, cultivar-specific signature that merits future investigation to determine its specific functional role in the postharvest biology of *F. filiformis.*

Crucially, this signaling signature in the SB cultivar coincided with a distinct energy metabolism profile and a significant decrease in ATP content (Figure 4d). In postharvest physiology, the maintenance of cellular energy homeostasis is widely recognized as a critical determinant for delaying deterioration and browning [22]. Therefore, we propose a testable, integrative model: the differential phosphorylation-mediated signaling landscape (involving nodes like Ras1 GEF/MAPK) may contribute to an inefficient metabolic state, and the resulting energy deficit could be a key convergent factor associated with the accelerated browning phenotype. The specific functional impact of the identified phosphosites and the causal links within this proposed network remain to be experimentally validated.

### 4.3. Energy Metabolism Dysregulation and Its Correlation with ATP Depletion

Energy metabolism is pivotal for the postharvest quality of horticultural products [23,24,25,26]. Our comparative analysis reveals that its regulation is closely linked to browning susceptibility in *F. filiformis*. The identification of 50 phosphorylated proteins involved in energy metabolism and the construction of their interaction network (Figure 4a,b) reveal a complex regulatory landscape susceptible to post-translational control.

The cultivar-specific phosphorylation profiles of key enzymes (Figure 4c) point to divergent regulatory states between the SB and RB cultivars. Critically, these molecular differences coincide with physiological outcomes. The significant decrease in ATP content exclusively in the SB cultivar (Figure 4d) strongly correlates with its more severe browning phenotype. This ATP depletion signifies a critical energy crisis that contributes to the loss of cellular homeostasis. Our observation aligns with and extends the established paradigm across postharvest systems: in longan fruit, energy deficit driven by metabolic dysfunction leads to pulp breakdown [24]. Meanwhile, ameliorating the energy deficit is central to maintaining the quality in strawberry [27]. More broadly, adequate intracellular ATP is recognized as a fundamental determinant for delaying senescence and attenuating postharvest stress across horticultural crops [28]. Thus, the energy crisis in SB may severely impact the phosphorylation-dependent regulatory networks highlighted earlier (Figure 4c), locking the cells into a state of dysregulated signaling and failed stress adaptation.

### 4.4. Predicted Kinase Families and Their Potential Roles in Postharvest Signaling

Our kinase prediction analysis revealed that the SB cultivar is characterized by a higher relative abundance of predicted kinases from several major families (Figure 5). This finding suggests that the differential phosphoproteome of the SB cultivar may be influenced by distinct kinase signaling landscapes.

Notably, predicted kinases from the CMGC (including MAPK and CK2), AGC, CAMK, and CK1 families were predominantly more abundant in the SB cultivar. These kinase families are known in various biological contexts to be involved in the regulation of cellular growth [29], senescence [30], and stress response [31], consistent with their potential engagement in postharvest deterioration. For instance, the MAPK signaling pathway is a well-conserved module for transducing extracellular stresses, including oxidative stress, into intracellular responses [5]. The generation of ROS in postharvest *F. filiformis* could therefore involve MAPK pathway activity, which in other species regulates downstream senescence-related gene expression, as shown in tomato, papaya, and banana [8,9,32]. Furthermore, the application of chitosan-based packaging in *A. bisporus* was shown to enhance storage stability by upregulating MAPK pathway genes [10], underscoring the potential relevance of this kinase family in the postharvest biology of fungi.

Beyond MAPKs, the elevated abundance of other predicted kinase families points to a broader shift in the signaling environment of the SB cultivar, likely reflecting an intensified effort to cope with postharvest stress. Kinases such as GSK3, AGC, and CAMKLs are extensively studied in animal systems as central regulators of metabolism and stress signaling [33,34]. For instance, AGC family kinases like AMPK and PKB/Akt are pivotal energy sensors that modulate autophagy and cell survival under energy deficit—a state mirroring the ATP depletion we observed in SB [35]. Similarly, in plants, GSK3 kinases are key negative regulators of growth that are suppressed under stress to promote resilience, while CAMKLs often mediate calcium-dependent stress responses [36]. The concerted prominence of these kinase families in our prediction data strongly positions them not merely as generic stress markers but as candidate master regulators within a rewired signaling network that potentially governs the accelerated senescence trajectory in edible fungi postharvest.

### 4.5. Integration of Predicted Kinase Activity with Energy Metabolism Alterations

The PPI network analysis offers an integrated view that links the predicted kinase activity with the energy metabolism alterations observed in the browning-sensitive SB cultivar. It highlights potential regulatory nodes that could concurrently affect multiple facets of energy metabolism.

The finding that a single predicted CAMK kinase (EVM0004287) is associated with both ATP synthesis (via ATP synthase), ATP degradation (via E1-E2 ATPase), and electron transport chain integrity (via SQR) suggests a potential point of coordinated regulation over cellular energy charge (Figure 6a). This aligns with established roles of Ca^2+^-dependent kinases in modulating mitochondrial function [37,38]. In the postharvest context of *F. filiformis*, we hypothesize that the differential regulation of this CAMK node might be linked to the disruption of ATP turnover balance. This model provides a plausible explanation for the rapid ATP depletion observed in the SB cultivar (Figure 4d) and is supported by prior evidence that ATP depletion is a critical event in postharvest deterioration of related species [39].

Furthermore, the network indicates that the predicted CK1 kinase (EVM0002922.1) is associated with the key antagonistic enzymes 6-phosphofructokinase (PFK, glycolysis) and fructose-1,6-bisphosphatase (FBPase, gluconeogenesis), suggesting its potential role in modulating glycolytic flux (Figure 6b) [40]. A critical, unresolved question is whether CK1-mediated phosphorylation in the SB cultivar would promote a futile cycle that wastes ATP or lead to inefficient glycolytic activation. This represents a compelling focal point for future research.

While these regulatory paradigms are well-characterized in mammalian systems [37,38,40], their roles in fungal postharvest biology are largely unexplored. Our analysis proposes that these predicted kinase-energy metabolism interactions constitute a testable model for how differential signaling could govern the efficiency of cellular energy production and utilization, thereby influencing postharvest resilience.

## 5. Conclusions

Our comparative phosphoproteomic analysis reveals that the rapid postharvest deterioration of the SB *F. filiformis* cultivar is associated with a distinct, pre-existing phosphorylation signature in its signaling landscape. This signature features an elevated abundance and predicted activity of kinases such as CAMK and CK1, which are linked to alterations in both ATP synthesis and glycolytic pathways. The resultant correlated inefficiency in energy flux provides a coherent model to explain the severe ATP depletion and loss of energy homeostasis that are associated with this cultivar’s susceptibility. Collectively, our data support a model where the inherent shelf-life limitation may arise from a phosphorylation-mediated disturbance in energy homeostasis under postharvest stress.

## Data Availability

The mass spectrometry proteomics data have been deposited to the ProteomeXchange Consortium (https://proteomecentral.proteomexchange.org, accessed on 5 November 2025) via the iProX partner repository with the dataset identifier PXD0663300.The raw data were processed using DIA-NN software (version 1.8.1) as detailed in the Methods section (Section 2.5).

## Abbreviations

The following abbreviations are used in this manuscript:

SB	Susceptible-to-browning
RB	Resistant-to-browning
DIA	Data-independent acquisition
PPI	Protein–protein interaction
ROS	Reactive oxygen species
MAPK	Mitogen-activated protein kinase
GPx	Glutathione peroxidase
PSMs	Peptide spectrum matches
FDR	False discovery rate
FC	Fold change
DPPs	Differentially regulated phosphoproteins
iRT	Internal retention time
GO	Gene Ontology
KEGG	Kyoto Encyclopedia of Genes and Genomes
PCA	Principal component analysis
PHK	Phosphorylase kinase
GSK3	Glycogen synthase kinase 3
CAMKL	Calcium/calmodulin-dependent protein kinases
